# Supplementary material for: Are carbon emissions trading and green financial instruments synergistic? -Comprehensive quantitative research based on content analysis
Source: PLoS One. 2024 Mar 7;19(3):e0298601. doi: 10.1371/journal.pone.0298601 (PMC10919601; doi:10.1371/journal.pone.0298601)
Supplement: S1 Data — (ZIP) [file pone.0298601.s001.zip › Data/地级市Z_F工作报告.zip!/╣π╬≈/╣≤╕█╩╨/└┤╫╘╣1⁄2╓┌║┼í╛┬φ┐╦╩2╛▌═°í┐_2012.docx]

现在，我代表市人民政府向大会作政府工作报告，请予审议，并请列席会议的政协委员和其他同志提出意见。

2011年工作回顾

刚刚过去的2011年，是实施“十二五”规划的开局之年。一年来，在自治区党委、政府和市委的正确领导下，在市人大及其常委会、市政协的监督支持下，我们紧紧依靠全市人民，坚持以科学发展观统领全局，以建设西江流域核心港口城市为目标，以实施“三百工程”项目建设大会战为载体，全力以赴抓发展，千方百计保民生，经济社会实现了平稳较快发展。预计全年实现地区生产总值631.7亿元，增长7%；财政收入43.3亿元，增长8.2%；全社会固定资产投资443亿元，增长15％；外贸进出口总额2.6亿美元，增长70.2%；实际利用外资2.1亿美元，增长34.6%；城镇居民人均可支配收入17084元，增长10％；农民人均纯收入6505元，增长23%。各项社会事业协调发展。

一年来，我们着重加大了以下六方面工作的力度：

（一）凝心聚力抓发展，促进经济保持平稳增长。面对国内外严峻的经济形势和持续性的自然灾害，我们认真贯彻落实中央和自治区的一系列政策措施，加强分类指导，强化要素保障，努力克服各种不利因素，经济发展呈现良好态势。三次产业的比重由2010年的19.8:45.6:34.6调整为19.2:46.8:34，产业结构进一步优化。工业经济运行良好。预计全市规模以上工业总产值560亿元，增长19.1%；上缴税金19亿元，增长25.1%。新增规模以上工业企业12家，新增年产值超亿元企业10家，新增纳税超千万元企业4家。技术改造步伐加快，企业完成更新改造投资98亿元。自治区下达的节能减排项目建设全面完成。农业经济平稳发展。预计农林牧渔业总产值208.8亿元，增长3.5%。粮食生产稳定增长，预计总产量达146.1万吨，增长3.9%；优势特色种养业规模扩大，新增高效优势农业面积15.3万亩，新增规模养殖场（区）320个。农业产业化进程加快，新增市级农业龙头企业10家、农村专业合作社90个。“菜篮子”工程建设取得初步成效。集体林权制度改革深入推进。农业综合机械化水平稳步提高。第三产业快速发展。预计第三产业增加值214.7亿元，增长7.4%。商贸流通业日益繁荣，实现社会消费品零售总额243.8亿元，增长17.7％。港口货物吞吐量达到4100万吨，增长7.6%；其中集装箱吞吐量9万标箱，增长27.62%。旅游业加速发展，旅游接待人数和总收入分别增长21%、40%。金融、保险、证券、餐饮等服务业发展加快。

（二）抢抓沿江开发战略机遇，西江经济带规划建设有序推进。认真实施港口、城市、园区互动发展战略，沿江开发建设成绩斐然。港口规划进一步深化，沿江107公里可利用岸线、西江经济带产业、沿江物流系统综合开发以及沿江重点作业区的控制性详规取得阶段性成果。沿江基础设施建设步伐加快，贵港至南宁二级航道开工建设，进港公路、猫儿山作业区二期工程等重大项目顺利推进，桂平二线船闸竣工通航。沿江产业园区建设成效显著，全市五大产业园区总体规划和核心区控制性详规基本完成，园区管理体制、考核办法以及园区道路、供水、供电等基础设施进一步完善，从而有力地推动了园区加快发展。预计全市五大产业园区实现工业增加值71亿元，增长19.7%；上缴税金10亿元，增长38.8%。

（三）狠抓项目建设，着力增强经济发展后劲。坚持把抓投入、抓项目作为加快发展的突破口，进一步健全重大项目推进机制和督查考核机制，深入实施“三百工程”项目建设大会战，有力地推动了全市投资稳定增长。全年实施规模以上主要建设项目738个，完成投资295亿元。大藤峡水利枢纽工程进坝公路、江南污水处理厂等一批重大项目实现开工，中联陶瓷一期、巴帝食品二期等一批重大项目实现竣工投产，新权业陶瓷二期、永源生物科技等重大项目有序推进，大藤峡水利枢纽工程项目获得国家发改委批复立项。新引进了益海嘉里粮油加工、隆昌纺织等一批重大产业项目。

（四）扎实推进城乡建设，城乡基础设施进一步完善。立足于城乡统筹，大力加强基础设施建设，着力优化人居环境。坚持以规划引领建设，认真编制了中心城区六大片区控制性详规和城市设计以及城区道路、排水等专项规划。中心城区的重点市政工程建设有序推进，建设路西延段、南山路等城市道路以及城市绿化、亮化、美化工程建设进度加快，市图书馆实现竣工，市博物馆完成主体工程。市文化艺术中心、市体育中心、市中心医院等项目基本完成规划设计、定点选址，目前正在进行项目征地和工程招投标。深入开展市容市貌综合整治和违法用地建设查处工作，城市环境有所改观。投入30多亿元建设城乡公路，梧州至贵港、桂平至来宾高速公路和南广高速铁路（贵港段）、东环郁江大桥工程加快推进，建设农村公路450公里；投入6.5亿元加强农田水利基础设施建设，完成病险水库除险加固16座、渠道防渗334.8公里，新增灌溉面积4.1万亩；投入5.08亿元用于电力设施建设，一批重大电网项目实现竣工。

（五）统筹经济社会发展，各项社会事业全面进步。坚持统筹兼顾，大力发展各项社会事业。优化教育资源配置，多渠道筹资1.59亿元投入校安工程建设，改造校舍面积13.3万平方米；中心城区的新建荷城初中和县西小学扩建一期、峡山初中扩建工程均竣工投入使用。学前教育规模进一步扩大，义务教育得到巩固提升，高中教育质量稳步提高，职业教育攻坚通过自治区评估验收。稳步推进基层医药卫生体制综合改革，基层医疗卫生机构实施基本药物制度实现全覆盖；新型农村合作医疗制度建设取得新突破，参合率达95.8%；完成7个乡镇卫生院改扩建和1073个村卫生室规范化建设。不断增强科技创新能力，新增高新技术企业1家。文化事业健康发展，新建乡镇综合文化站7个、村级公共服务中心42个、农家书屋635个。全民健身活动深入开展。建立完善人口和计划生育长效工作机制和利益导向机制，建成243个人口计生“幸福家园”村（居）级综合服务平台。扎实推进“双拥”工作，荣获自治区“双拥”模范城“八连冠”称号。统计调查、气象服务、防震减灾、外事侨务、民族宗教、民兵预备役、地方志、住房公积金和妇女儿童、老年人、青少年、残疾人等事业都取得了新成绩。

（六）坚持以人为本，民生保障得到进一步加强。坚持把保障和改善民生作为政府一切工作的出发点和落脚点，加大民生实事的投入，财政用于解决涉及民生事业的支出占全市一般预算支出的74.5%。稳步推进就业再就业工作，新增城镇就业1.78万人，城镇登记失业率为3.72%，新增农村劳动力转移就业4.5万人。着力扩大社会保险范围和规模，社会保险参保人数达154.3万人次，比上年增加29.8万人次。进一步加强城乡低保、医疗、五保等社会救助和救灾救济工作，共投入救助资金4亿多元，救助各类对象400多万人次。第三批整村推进扶贫工作通过了自治区验收，全市减少贫困人口3.5万人。着力推进保障性安居工程，开工建设保障性住房5826套，棚户区改造1018户，农村危房改造8500户，发放租赁补贴5001户，有效解决了市民住房困难和住房条件差的问题。投入2.15亿元实施农村安全饮水工程223个，受益农民43.95万人。投入2600多万元，新建沼气池6200座。及时采取临时价格干预措施，通过供应平价大米、限价销售猪肉和食用油，对困难群体发放临时价格补贴，有效的平抑了物价上涨势头。加强安全生产管理，各类事故得到有效控制。切实做好来信来访和矛盾化解工作，大力完善社会治安防控体系，严厉打击各类犯罪行为，社会和谐稳定。

在积极推进经济社会发展的同时，我们更加注重政府自身建设，政府执行力进一步提高。深入推进依法行政，严格规范行政行为。自觉接受人大和政协监督，办理人大代表建议57件、政协委员提案227件，办结率100%。加强电子政务建设，主动公开政府信息，建立和完善了土地招投标、建设用地改变土地使用条件管理办法等制度，政府公信力和透明度进一步提高。深入推进扩权强县改革，下放管理事项105项。加强政风行风建设，不断完善政府绩效考评体系，优化行政审批流程，行政效能有所提高。全面落实行政执法责任制，完善行政问责制度。严格落实行政监察、审计监督、财政监管和“一岗双责”廉政建设责任制，严肃查处各种违纪违法案件。

各位代表，过去的一年，在宏观经济环境发生重大变化，发展的不确定因素明显增多，组织运筹经济工作难度不断加大的情况下，我们能够保持良好的发展态势，成绩确实来之不易。这些成绩的取得，是在市委的正确领导下，在市人大、市政协的支持和监督下，全市人民团结拼搏、攻坚克难、开拓创新的结果。在此，我代表市人民政府向全市各族人民，向各民主党派、工商联、人民团体和各界人士，向驻贵中区直单位和驻贵部队、武警官兵，向所有关心支持贵港发展的朋友们，表示诚挚的感谢和崇高的敬意！

在肯定成绩的同时，我们也清醒地看到，就总体发展水平而言，贵港在全区的排位仍然比较靠后，经济总量小、基础差、底子薄、人均水平低的现状还没有根本改变，加快发展的任务还十分艰巨。特别需要引起高度重视的是，去年我市地区生产总值、财政收入、城镇居民人均可支配收入等一些主要指标未能完成预定计划，一些重大项目未能如期组织实施。年初的预期目标，虽然是指导性、预测性的，但经济增长速度的回落，这就暴露出我市经济结构和经济运行中的薄弱环节。究其原因，既有国内外大环境的不利因素影响，也同政府自身工作的不足密切相关：一是受国际金融危机、国家宏观调控政策等综合因素影响，以资源型为主的单一经济结构矛盾更为突出，大部分企业因成本增加、融资困难、用电紧张等生产经营问题而出现限产、停产，直接影响了地区生产总值、财政收入等主要预期目标的实现。二是经济发展缺乏龙头企业引领和大项目带动，企业低小散状况没有根本转变，经济增长的内生动力不足，抗风险能力和产品竞争力较弱，导致财政缺乏稳定的支撑。三是产业招商缺乏有效举措，对事关全局的重点项目谋划不够，项目储备不足，前期工作和基础性工作不扎实，致使一些重大项目的引进和建设没有达到预期目标。四是从政府自身建设方面看，经济和社会管理的水平还需要进一步提高，个别部门和少数干部思想解放不够、观念更新不快、工作作风不实、服务效率不高等现象仍然存在。对于这些困难和问题，我们将积极面对，采取有效措施，认真加以解决，不辜负全市人民的重托和期望。

2012年工作安排

2012年是承前启后、实现“十二五”跨越发展目标的关键一年。当前贵港正处于新一轮加快发展的关键时期，标兵越来越多、追兵越来越近，形势逼人。面对各地抢抓机遇、竞相发展的态势，贵港必须奋起直追，实现跨越式发展。根据贵港市第四次党代会精神和我市“十二五”规划确定的发展目标，市委、市政府决定实施港口、城建、园区、农业、民生等重点工作三年发展目标任务行动计划，通过把经济社会发展任务实行项目化管理，靠项目的攻坚，带动经济社会又好又快发展，努力实现“一年有突破、两年上台阶、三年见成效”。如果今年的攻坚目标不能如期实现，势必影响“十二五”规划任务的完成。因此，做好今年的工作，保持经济社会健康较快发展的良好势头，意义十分重大。全市上下务必保持蓬勃向上的朝气、敢闯敢试的勇气和开拓进取的锐气，抢抓新机遇，创造新业绩，推动大发展。

2012年政府工作的总体要求是：以邓小平理论和“三个代表”重要思想为指导，以科学发展观统揽全局，认真贯彻落实中央、自治区经济工作会议精神和自治区第十次党代会、贵港市第四次党代会精神，深入推进港口、城市、园区互动发展战略，大力实施“港口、城建、园区、农业、民生”三年发展目标任务行动计划，努力实现经济质量有新提高、城市面貌有新改变、社会管理有新提升、人民生活有新改善、和谐社会建设取得新突破，全力打造西江流域核心港口城市。

2012年经济社会发展的主要预期目标是：地区生产总值增长11％，全社会固定资产投资增长16％，财政收入增长12％，规模以上工业增加值增长17％，社会消费品零售总额增长15％，进出口贸易总额增长20％，实际利用外资增长15%，城镇居民人均可支配收入增长11％，农民人均纯收入增长12％，居民消费价格总水平涨幅控制在5％左右，城镇登记失业率控制在4%以内，人口自然增长率控制在9‰以内，全面完成自治区下达的万元地区生产总值能耗、主要污染物减排量等指标。在实际工作中，我们将遵循经济发展稳中求快、重在加快、努力实现又好又快的原则，适时调整，力争超过预期目标。

为实现今年目标任务，在全面抓好各项工作的同时，重点在以下六个方面实现新突破：

（一）着力完善基础设施布局，在打造现代大港口上实现新突破

紧紧围绕建设西江流域核心港口城市目标，以勇当全区西江经济带开发排头兵为定位，以西江经济带基础设施建设大会战为抓手，强力推进沿江开发攻坚突破，把资源优势转化为发展优势和竞争优势，全面提升沿江开发的层次和水平。年内组织实施19个重点项目，计划完成投资7.95亿元。

完善港口发展规划。坚持以规划引领开发，加快修编和完善沿江107公里可利用岸线、港口新区、公共作业区等重点区域的控制性详规，深化港区水、电、路等专项规划，完成《贵港市西江经济带产业发展规划》、《贵港市港口岸线及物流系统综合开发概念性规划》等专题性规划编制工作。在加强各类规划编制和衔接完善的同时，切实抓好港口岸线资源和土地资源的保护管理。

强力推进港区设施建设。开工建设贵港航运枢纽二线船闸、苏湾作业区一期、棉宠作业区一期、武林港二期、猫儿山作业区东山多用途泊位工程，加快推进罗泊湾扩建20万标箱及件杂货配套堆场和库场、猫儿山作业区二期、郁水作业区永泰码头等项目建设，配合推进大藤峡水利枢纽、南宁至贵港2000吨级航道等项目建设，认真做好贵港至梧州3000吨级航道和来宾至桂平2000吨级航道等项目的前期工作。加快沿江进港公路、铁路的工程建设，逐步形成集公路、铁路、水路为一体的疏运体系。完善口岸配套功能，优化口岸通关环境，提高通关效率。年内新增港口货物吞吐能力600万吨以上。

加固基础设施支撑。配合加快推进梧州至贵港、桂平石龙至来宾、梧州至柳州高速公路和南宁至广州高速铁路（贵港段）建设，年内力争开工贵港至合浦、荔浦至玉林高速公路（贵港段），强化区域路网互联互通。进一步深化与广西西江投资集团的战略合作，积极吸引具有雄厚实力的大型水运企业、修造船企业和投资开发企业落户贵港。强化政策和重大项目与国家、自治区的对接，争取更多的项目列入自治区“西江经济带规划”或专项规划。围绕增强口岸开放功能，积极筹备申报贵港港保税仓，继续努力争取设立贵港保税港区。

积极发展现代服务业。进一步整合港口、铁路站点、高速路口以及沿江产业园区等物流资源，切实做好重点物流园区和物流业态的规划、招商、建设工作，重点推进煤炭、水泥、钢材、家居建材、木制品、农副产品、粮糖、日用品等物流园区或专业市场的规划建设。年内力争开工建设商业中心、汽车服务贸易园、煤炭交易市场、钢材交易市场、小商品城等一批综合物流商贸项目，完善主城区核心商圈功能，不断聚集城市的商气人气财气。积极引进各类国内外知名物流企业到贵港设立区域总部或办事机构，带动中小物流企业发展。大力提升传统服务业层级，加快发展新兴服务业。要以更大的力度加快发展旅游业。进一步整合旅游资源，加强旅游产品开发和市场营销，优化旅游线路。年内编制完成贵港市旅游总体规划，探索组建跨地区、跨行业的旅游企业集团，积极引资建设九凌湖旅游度假区、八桂文化生态旅游产业园区、白石山旅游区、鹏山人文生态旅游区等特色旅游项目。力争全年旅游接待人次和旅游收入分别增长10%、12%以上。

（二）加强载体建设和产业集聚，在增强园区承载能力上实现新突破

全面加大园区基础设施建设力度，把做优载体作为促进招商引资上项目、打造沿江大产业的重要保障。年内重点组织实施43个工业投资项目和14个基础设施建设项目，计划投资37亿元。

强化园区功能配套建设。按照“竞争力强、集约度高、配套性好”的要求，进一步完善园区发展规划、建设详规和功能分区。坚持政府主导、市场运作、多元投入，加快标准化厂房和污水处理厂、公租房、路网等园区基础设施建设，进一步拉伸框架，完善功能，集聚项目。着力完善园区管理机制、监督机制、考核机制和激励机制，严格执行入园项目评审和不良项目退出管理制度，加强开发建设效益核算，不断提高整体开发水平。

加快推动产业集聚。围绕园区产业定位和五大支柱产业培育，抓好重点企业引进、主导产业培育、重大项目建设和龙头企业发展，重点鼓励和支持财税型、基地型企业在规划区域自建产业园，打造规划科学、综合利用的生态工业园中园，形成聚合效应。市江南工业园重点推进皮革城、隆昌纺织园等项目；贵港国家生态工业（制糖）示范园区重点推进华劲纸业、香港茂荣毛织基地等项目；贵港（台湾）产业园重点推进粮糖物流园、海都陶业、润桂重工修造船等项目；桂平长安工业园重点推进龙门陶瓷城、船舶修造、金源生化技改等项目；平南工业园重点推进美的三竹电器、天保生物科技等项目。力争年内每个产业园区至少引进一个纳税超亿元的产业项目，规划建设好一个面积1500-2000亩、基础设施比较完善、产业项目较为集中、企业成长性较好的产业核心区或特色功能区。

突出扶优培强。按照因地制宜、分类指导的原则，坚持抓大壮小扶微，在项目申报、土地供应、融资贷款、鼓励上市等方面加强扶持龙头企业、骨干企业和成长性较好的企业，不断扩大规模企业阵容。年内新增规模以上企业20家以上，新增年产值超亿元企业5家以上，力争新增一个产值超百亿元的支柱产业。大力发展直接融资，加快培育上市后备资源。积极发展绿色经济、循环经济，推动企业节能减排技术改造和产品升级换代，确保全面完成节能减排任务。

（三）强化城市规划建设和管理，在提升城市品位上实现新突破

围绕港口、城市、园区互动发展，加快完善城市综合服务功能，提升城市形象和品位。年内组织实施69个重点项目，计划投资70亿元。

高起点规划城市。按照“规划先行、适度超前”的原则和“城市布局科学化、建筑风格特色化、居住环境生态化、综合功能配套化”的要求，高品位完善中心城区城西片区、港口新区、城北新区二期、郁江北堤片区、火车站片区、江南片区等六大组团以及沿江12个重点镇的控制性详规，编制城市交通、管网、绿地、商业网点、公益设施布局、城市地下空间利用、郁江滨水环境景观等专项规划，努力使规划与城市分区功能定位、资源环境和产业基础相协调，提高规划的科学性、前瞻性和可操作性。继续做好小城镇和乡村规划编制工作。

高标准建设城市。坚持分片区、分组团推进新区建设和旧城改造，大力拓展城市规模。围绕六大片区规划，按照“东西扩张、南北拓展、中间突破”的思路，组织实施城市建设大会战。进一步完善中心城区路网。开工建设金港大道东延段、解放路北延段、布山大道、江滨大道等24条（段）城市道路，加快推进东环郁江大桥、同济大道和解放路下穿铁路工程等项目建设，规划建设同济大桥、西南大桥、贵港至覃塘一级公路，缓解城区交通拥堵状况。积极开发利用城市地下空间，打造立体交通体系。进一步优化和完善公共设施服务体系。开工建设市文化艺术中心、体育中心和中心医院、精神卫生医院，建成市档案馆和博物馆。加大老城区改造和建成区配套设施建设力度，继续实施郁江北堤堤路园、东湖综合整治和城区背街小巷整治工程；加快市区农贸市场、新火车站广场、商场、学校等公共服务设施建设，进一步增强城市综合服务功能。力争年内中心城区新增建成区3.2平方公里。各县城都要立足特色定位，加快老城改造和新区整体联动发展步伐，完善提升县城公共服务和居住功能。着力推进沿江12个重点城镇、名镇名村工程建设，进一步完善功能、扩大规模，更好地发挥中心集镇的要素集聚和辐射带动作用。力争年内城镇化率提高1.5个百分点。

高水平管理城市。严格规划管理，加强过程控制，坚决查处违法违规用地和建设的行为，切实维护规划的连续性、权威性和严肃性。坚持严管重罚和教育疏导相结合，健全完善城市管理长效机制，提高城市管理效能。大力实施城市绿化、亮化、净化、美化和畅通工程，加大城市环境整治力度，着力解决城市“脏、乱、差”的突出问题。年内重点实施南山景区扩建、民族文化公园、园博园等工程，认真抓好街景综合改造和夜景亮化，不断提升城市品位。加强社区环境和小区物业管理，健全社区自我服务体系，推进和谐社区创建。统筹规划生活、医疗和建筑垃圾处理设施建设，重点加快推进广西（桂东南地区）循环经济环保产业示范基地建设，实现以垃圾焚烧发电为核心的多种废弃物综合处理和循环利用。

高效能经营城市。进一步发挥建投公司的作用，做实做强投融资平台，有效运作城市和土地资本，增加收益。探索建立城市资产转让、拍卖、租赁、置换的有效途径，推进城市资源资本化、资源配置市场化。积极鼓励金融资本和民间资本多渠道、全方位参与城市开发，全面提升城市建设的市场化、资本化运作水平。严格土地投资强度、建筑容积率等指标，规范土地征用、拆迁和建设管理。认真贯彻国家房地产调控政策，促进房地产业健康稳定发展。

（四）大力发展农业产业化经营，在推进现代农业建设上实现新突破

坚持以工业的理念发展农业，按照“工业化致富农民、城镇化带动农村、产业化提升农业”的基本思路，稳步推进农村改革发展，加快发展农村公共事业，不断促进农业增效、农民增收、农村繁荣。年内组织实施125个重点项目，计划投资10.5亿元。

积极推进农业产业化。坚持“集约化、标准化、产业化”的思路，大力实施农户“万元增收工程”和“菜篮子”工程，以“一村一品”、“一乡一业”为载体，加快培育一批专业村、特色镇和高效农业基地，促进特色优势产业快速发展，年内新增高效农业面积5万亩，新建扩建规模养殖小区（场）500个。采取财政贴息、以奖代补、小额信贷、互助合作等方式，引导农民专业合作社健康发展，引进和培育一批产业规模大、带动辐射能力强的种养型、加工型、流通型龙头企业。年内新增农民专业合作社40家、市级以上农业产业化龙头企业5家。继续实施“万村千乡”工程和“新网”工程，加强县乡配送中心、综合超市、农贸市场等农村流通体系建设，大力推行“农超对接”和“农市对接”，拓宽农产品销售网络和渠道。

努力改善农村生产生活环境。抓住国家加大水利投入的政策机遇，加强以农田水利、饮水安全为重点的农村基础设施建设，提高农业综合生产能力。年内完成35座病险水库除险加固，新增、恢复或改善灌溉面积10万亩，解决43万农村人口饮水安全和困难的问题。继续大力推进农村公路建设，新建农村公路300公里，通村水泥路比例达78%以上。加快农村改圈、改厕、改厨，新建户用沼气池4000座以上。统筹推进扶贫开发和移民扶持工作，年内实现农村脱贫人口7.5万人，建成39个移民新村。深入推进以城镇绿化、通道绿化、村屯绿化为主的“绿满八桂”造林绿化工程，切实改善生态环境。扎实推进城乡风貌改造工程，着力推广农村垃圾“村收集、镇清运、县处理”模式，抓好农村环境连片综合整治，进一步改善农村人居环境。

扎实推进农村改革。全面落实各项强农惠农政策，增加对农业基础设施和农村公共事业的投入。积极创新农村土地流转机制和流转平台，支持和引导农民通过转包、出租、互换、转让、股份合作等多种形式，流转土地承包经营权，增加农民土地收益。进一步深化水管体制改革和集体林权制度配套改革。加大科技兴农力度，完善农技推广和服务体系。着力提高农业综合机械化水平。加快建设市、县两级农产品质量安全检验检测中心，健全完善农产品质量安全监管体系。创新金融支农机制，满足农村金融服务需求。落实政策性农业保险制度，探索建立农业灾害补偿机制，提高农业风险保障能力。

（五）着力加强和改善民生，在提升公共服务水平上实现新突破

顺应人民群众过上更加美好生活的新期待，以解决人民群众最关心、最直接、最现实的利益问题为重点，更大力度落实各项惠民举措，更大力度推进民生工程建设，努力让人民群众分享更多发展成果。年内组织实施259个重点项目，计划投资25.7亿元。

进一步完善社会保障体系。建立健全城乡统筹就业机制，加强就业技能培训，以创业带动就业。力争年内新增城镇就业1.6万人，新增农村劳动力转移就业4万人。健全城乡社会保障体系。继续巩固和扩大各类社会保障制度覆盖面，重点推进灵活就业人员、农民工等参加城镇职工基本养老保险，努力实现城乡居民基本医疗保险、基本养老保险和新农合制度全覆盖。落实征地补偿政策，保障被征地农民基本生活。继续完善优抚安置保障体系和社会救助体系，加大城乡社会福利设施建设力度，年内新建一批五保村，规划建设集儿童福利院、老年人活动中心、福利医院、救灾物资储备仓库为一体的贵港市社会福利中心，在中心城区和桂平、平南分别规划建设一所公办残疾人集中托养机构。完善城乡居民低保、农村五保户、被征地农民生活保障标准自然增长机制。加强法律援助，保障困难群众合法权益。完善保障性住房准入制度、退出制度和信息管理制度，加大以公租房为主的保障性住房建设。年内建设保障性住房6000套以上，完成农村危房改造9000户以上。积极发展慈善事业和老龄事业，努力提升民生保障水平。

优先发展教育事业。多渠道加大教育投入，促进各类教育协调发展。进一步优化中小学布局，加快中小学校舍改造和寄宿制学校建设，不断改善办学条件。按照教育发展规划及布局结构调整规划，年内在中心城区新建一所小学、一所初中和改扩建6所中小学校。大力发展学前教育，年内建设乡镇公办幼儿园30所以上。全面完成中小学校舍安全工程三年规划目标，启动义务教育规范化学校建设和农村艰苦地区学校教师周转房建设。提高高中阶段教育普及水平，推动普通高中多样化和特色化发展。大力支持民办教育发展，满足人民群众对教育的多样化需求。加快职业教育资源整合，开展职业教育创新办学模式，提高教育服务经济社会发展能力。支持贵港职业学院发展壮大，启动广西医科大学贵港校区创建工作，鼓励和支持区内外优质高校到贵港合作办学。重视发展特殊教育，规划建设中心城区和桂平、平南三所特殊教育学校，为残障儿童营造良好的教育环境。继续加强学生资助工作，切实解决家庭经济困难学生“上学难”的问题。加强教师队伍建设，提升教师业务素质和职业道德水平。

促进文化繁荣发展。加快重点公共文化基础设施建设，新建60个村级公共服务中心。继续大力实施广播电视村村通工程、文化信息资源共享工程、农家书屋工程、农村电影放映工程等文化惠民工程，切实提高城乡公共文化服务水平。加大文物保护力度，加强文化遗产和非物质文化遗产的保护、开发与利用。充分挖掘历史文化、民族文化等文化资源内涵，大力倡导“和为贵”的价值理念，唱响“和谐在贵港、贵港最和谐”的主旋律，努力培育“和为贵”的贵港文化品牌。发展壮大广播电视、出版发行、演艺、印刷等传统产业，大力发展文化生态旅游业，培育发展新型文化业态。深化文化体制改革，增强文化实力和文化产业发展活力。把社会主义核心价值体系建设融入国民教育和精神文明建设全过程，加强社会公德、职业道德、家庭美德和个人品德建设，为实现跨越式发展提供思想道德保障。

协调发展科技、卫生等其他社会事业。认真实施第五轮创新计划，加强自主创新能力建设，建立自治区级创新型试点企业1家以上，企业开发工业新产品20个以上，争取认定高新技术企业1家以上。继续稳妥推进医药卫生体制改革，完善三级医疗服务网络，为群众提供安全方便的基本医疗卫生服务。重点抓好市中心医院和精神卫生医院的规划建设，加快改造县、乡、村和社区三级卫生医疗机构。启动县级公立医院改革试点工作。加强对重大传染病的防控，提高突发公共卫生事件应急处置能力。深入开展全民健身活动，推进乡村农民体育健身工程建设。大力推进“诚信计生”，统筹解决人口问题，稳定低生育水平。落实妇女儿童发展规划，切实维护妇女儿童合法权益。加大民族地区扶持力度，依法管理宗教事务，维护民族团结和宗教和谐。加强国防教育，提高国防动员、民兵预备役和后备力量建设质量。继续做好侨务、对台、地方志、住房公积金、防灾减灾等工作。

加强和创新社会管理。加强基层组织、社区的管理和服务设施建设，增强基层社区管理和服务能力。加强重点地区、重点人群、社会新经济组织和流动人口服务管理，强化对刑释解教人员和社区矫正人员等特殊人群管理、教育和帮扶。继续推进社会信用体系建设，健全个人和企业联合征信平台。加快建立网络管理机制，加强舆情监测和舆论引导，提高对虚拟社会的管理水平。建立健全群众诉求表达机制、社会矛盾调处机制以及重大工程项目建设和重大政策制定的社会稳定风险评估机制，着力从源头上预防和减少矛盾。坚持“属地管理”原则，落实信访工作责任制，妥善处理群众信访突出问题。加强价格监管，建立和完善价格调节基金管理机制，维护市场供应和物价基本稳定。强化应急指挥体系和应急救援队伍建设，提高应急救援和应急处置能力。抓好事关群众切身利益的农资、食品、药品等市场专项整治，保障人民群众生命财产安全。落实安全生产责任制，严防重特大事故发生。强化社会管理综合治理，不断完善以“维稳基石”工程为基础的“大防控”体系，严厉打击违法犯罪行为，增强人民群众安全感。

（六）切实加强政府自身建设，在提高政府执行力上实现新突破

对人民负责是政府工作的第一准则，让人民满意是政府工作的第一追求。市政府将努力探索并促进各级政府机关全体工作人员在新的历史条件下牢记宗旨，敢于负责，恪尽职守，改进作风，切实提高政府的执行力和公信力，努力建设人民满意政府。

坚持强化学习，以创新的思维做好工作。要在政府系统大兴学习之风、调研之风，提倡自觉学习，强化各类培训，不断提高广大公务员特别是各级领导干部的政治理论素养和开拓创新能力。引导各级干部紧紧围绕建设西江流域核心港口城市这个目标，进一步解放思想，转变观念，以敢为人先、勇于开拓的精神研究新问题、探索新规律，以科学的态度和创新的精神应对挑战、推动发展。只要有利于贵港科学发展，有利于人民群众的事情，就大胆地试、大胆地干、大胆地闯，以新理念引领新发展，以新举措激发新动力。

坚持依法行政，以完善的制度强化责任。各级政府和部门都要严格按照法定权限和程序行使权力、履行职责，坚决服从市委的领导，自觉接受人大依法监督和政协民主监督，认真听取各民主党派、工商联、无党派人士和各人民团体的意见，主动接受社会公众和新闻舆论的监督。严格执行行政执法责任制和过错责任追究制，完善行政执法监督制约机制。坚持公众参与、专家论证、风险评估、合法审查和集体讨论相结合，促进依法行政、科学施政。深化行政审批制度改革，完善电子监察系统，提高审批透明度和审批效率。加快建立和规范政府合同管理。加强政务公开和政府信息公开，健全完善政府工作运行机制，推进公共权力公开透明运行。

坚持务实高效，以过硬的作风推进落实。要坚持一切从实际出发，倡导简政、高效、务实的作风，多做打基础、管长远的工作，多办群众急需、便民利民的实事好事，坚决克服官僚主义、形式主义，戒空、戒虚、戒假、戒骄、戒懒、戒奢。进一步完善政府工作目标责任体系，健全重点工作推进责任机制和督办查办制度，对事关全局的重大决策，定时间、定标准、定要求，确保落实到位。进一步加强绩效考评工作，注重考评结果运用，严格兑现奖惩，强化激励导向，尤其要更加突出项目投入、绩效产出、财税贡献等考核权重，加快建立科学合理、行之有效的政府绩效考核评估体系。

坚持廉洁从政，以严格的自律树立形象。进一步抓好政府系统廉政建设，健全从源头上治理腐败的体制机制。加强对财政资金、重大投资项目、土地招拍挂、政府采购、国有资产资源经营管理等重点领域的行政监察和审计监督，真正做到用制度管权、管财、管事、管人。进一步增强各级政府工作人员廉洁自律的意识，大兴艰苦奋斗之风，努力节约行政成本，控制“三公”支出，把公共财政用在保重点、保运转、保民生上，努力树立为民、务实、清廉的政府形象。

各位代表，形势催人奋进，发展任重道远。让我们在市委的坚强领导下，紧紧依靠全市人民，倍加珍惜不可多得的历史机遇，倍加珍惜来之不易的发展势头，倍加珍惜安定团结的大好局面，与时俱进，乘势而上，扎实工作，为打造西江流域核心港口城市、实现富民强市新跨越而努力奋斗！
